# cgaTOH: Extended Approach for Identifying Tracts of Homozygosity

**DOI:** 10.1371/journal.pone.0057772

**Published:** 2013-03-01

**Authors:** Li Zhang, Mohammed S. Orloff, Sean Reber, Shengchao Li, Ye Zhao, Charis Eng

**Affiliations:** 1 Department of Quantitative Health Sciences, Lerner Research Institute, Cleveland Clinic, Cleveland, Ohio, United States of America; 2 Genomic Medicine Institute, Lerner Research Institute, Cleveland Clinic, Cleveland, Ohio, United States of America; 3 Taussig Cancer Institute, Cleveland Clinic, Cleveland, Ohio, United States of America; 4 Department of Medicine, Cleveland Clinic Lerner College of Medicine of Case Western Reserve University, Cleveland, Ohio, United States of America; 5 Department of Computer Science, Kent State University, Kent, Ohio, United States of America; 6 Department of Biostatistics, Johns Hopkins School of Public Health, Baltimore, Maryland, United States of America; 7 Stanley Shalom Zielony Institute for Nursing Excellence, Cleveland Clinic, Cleveland, Ohio, United States of America; 8 Department of Genetics and Genome Sciences, Case Western Reserve University, Cleveland, Ohio, United States of America; 9 CASE Comprehensive Cancer Center, Case Western Reserve University School of Medicine, Cleveland, Ohio, United States of America; Pennsylvania State University, United States of America

## Abstract

Identification of disease variants via homozygosity mapping and investigation of the effects of genome-wide homozygosity regions on traits of biomedical importance have been widely applied recently. Nonetheless, the existing methods and algorithms to identify long tracts of homozygosity (TOH) are not able to provide efficient and rigorous regions for further downstream association investigation. We expanded current methods to identify TOHs by defining “surrogate-TOH”, a region covering a cluster of TOHs with specific characteristics. Our defined surrogate-TOH includes cTOH, viz a common TOH region where at least ten TOHs present; gTOH, whereby a group of highly overlapping TOHs share proximal boundaries; and aTOH, which are allelically-matched TOHs. Searching for gTOH and aTOH was based on a repeated binary spectral clustering algorithm, where a hierarchy of clusters is created and represented by a TOH cluster tree. Based on the proposed method of identifying different species of surrogate-TOH, our cgaTOH software was developed. The software provides an intuitive and interactive visualization tool for better investigation of the high-throughput output with special interactive navigation rings, which will find its applicability in both conventional association studies and more sophisticated downstream analyses. NCBI genome map viewer is incorporated into the system. Moreover, we discuss the choice of implementing appropriate empirical ranges of critical parameters by applying to disease models. This method identifies various patterned clusters of SNPs demonstrating extended homozygosity, thus one can observe different aspects of the multi-faceted characteristics of TOHs.

## Introduction

Single nucleotide polymorphisms (SNPs), one of the most common variations across genomes, may present differently in human traits and disease susceptibility within and among populations. SNPs can function individually, but are frequently seen to work in coordination with other SNPs to manifest a disease condition, strongly indicating that much of the human diversity might be explained by larger structural differences between individual genomes, which are much larger than the single nucleotide differences. These structural polymorphisms include homozygous regions in the genome, e.g., Tracts of Homozygosity (TOH), which may have a significant role in both the genetics of complex diseases and genome evolution [1].

TOHs in study populations have been proven to be informative as covariates for association analysis with desired phenotypes. Moreover, homozygosity regions/loci have previously been shown to be a genomic risk factor for various disorders and cancers in both humans and animal models [2]–[7]. Recently, a number of studies have identified extended regions of homozygosity that are associated with specific cancers in unrelated individuals and individuals from consanguineous relationships [8]–[10]. Orloff *et al.* [11] have shown that these extended regions of homozygosity can act as novel genomic factors contributing to low to moderate penetrance predisposition to lung cancer. Current technologies, that allow studying multiple whole genomes simultaneously in many different individuals with dense SNP genotyping, provide a unique opportunity to examine the distribution, size and location of homozygous tracts and their relationship to biomedical outcomes.

Currently, the most popular software to identify TOH are the ROH module from Golden Helix [3],[12] and PLINK (–homozyg option) [13]. The ROH module of the Golden Helix software is designed to find runs of consecutive homozygous SNPs starting at every marker and to identify the location of those runs shared among the specified number of samples (common ROH), based on which, it also conducts whole genome homozygosity searches. However, the approach used to identify common ROH regions is not rigorous enough, since it often blends ROHs with distant boundaries in one common region. PLINK in contrast defines the ROH in terms of the required number of homozygous SNPs spanning a certain kb distance by the moving window approach and performs allelic-matching in overlapping pools. However, PLINK only supports very basic detection of long homozygous segments and lacks in providing the well-formatted results for downstream analysis.

To take full advantage of the TOH features and facilitate the downstream statistical analyses, our extended method focuses on newly-defined surrogate-TOH, which is a region covering a cluster of TOHs with specific characteristics. In the current paper, we introduce three types of surrogate-TOH, including cTOH, i.e., common TOH region sheltering a cluster of consecutive SNPs each belonging to a TOH across multiple subjects; gTOH, a region containing a group of overlapped TOHs with proximal boundaries; and aTOH, a region covering TOHs with overlapped allelically-matched regions. Searching for gTOH and aTOH is designed and implemented by repeated binary spectral clustering, a hierarchy of clusters represented by a TOH cluster tree. Towards an integrative analysis tool, the cgaTOH software is developed in a user-friendly command-line format, which also has the ability for an interactive visualization of TOH outputs and surrogate-TOH at the chromosome level or for individual surrogate-TOH and integrates visualization with statistical testing and NCBI genome map viewer for a better data understanding and decision making. We illustrate our method by utilizing a lung cancer dataset (part of PLCO, the Prostate, Lung, Colorectal and Ovarian cancer trial) obtained from dbGAP [14] and further discuss the potential range of crucial parameters based on the numeric results of various sets of parameters.

## Methods

### Identification Of Toh And Ctoh

For each subject, TOH is defined as a chromosome segment with consecutive homozygous genotypes residing on a single chromosome. Such a chromosome segment has a minimum length requirement, either with (1) a window of longer than consecutive SNPs or (2) a genetic distance longer than (kb) between the first and last SNP of the run (including a minimum number of SNPs). For illustration purposes, in the following, we mainly focus on a window specified by the number of SNPs. It allows a certain number of heterozygous and/or missing genotypes in a whole run and considers every potential run starting at every marker that meets the size (e.g., at least 100 SNPs) and density requirements. Common TOH (cTOH) is introduced as one type of surrogate-TOHs, in the sense that it is not a real TOH, it is rather a region where TOHs present relatively more frequently. We defined cTOH as a cluster of at least consecutive SNPs, each was contained in more than TOHs. For example, for each subject, a window of 100 or more consecutive SNPs on a single chromosome with homozygosity is defined as a TOH. Accumulation across all subjects is conducted for each SNP to determine whether a TOH is called at that position for a minimum number of individuals (e.g.,  = 10). cTOH then is identified as a window of 100 or more consecutive SNPs, each belonging to a TOH across more than 10 subjects. A subject whose individual TOH calls overlapped with a cTOH is called present for that cTOH.

### Searching Of Gtoh And Atoh

The cTOH region covers a cluster of consecutive SNPs which has a high likelihood of the presence of TOHs, but those TOHs do not all necessarily overlap, or their boundaries may be far from each other. Therefore, within a cTOH region, we apply repeated binary spectral clustering to group highly overlapped TOHs with proximal boundaries, termed as gTOH, in a pair-wise fashion. It facilitates finding more stringent regions covering the same extended homozygosity region. Specifically, gTOH is defined as a group of TOHs, among which the length of the overlap region for each pair is at least a certain percentage () of the length of each individual TOH. Since we are looking for a group of highly overlapped TOHs with proximal boundaries, is expected to be at least 50%. The range of a gTOH region is set as the lower quartiles of the start and end positions of all member TOHs, respectively, to avoid the extreme boundaries.

Homozygous for a SNP means both alleles at that SNP are identical, but it could be the homozygosity of either major or minor allele. Thus, the overlapping TOHs within a cTOH may or may not contain matched alleles. Therefore, within each cTOH, we are further looking for these allelic-matched groups of TOHs, i.e., aTOH. We also applied repeated binary spectral clustering, where the process is identical to the generation of gTOH regions with the exception of the similarity definition between each pair of TOHs. For the pairs whose overlap segments are longer than a certain percentage (e.g., 50%) of each individual TOH, allelic matching is performed across the overlap segment and similarity is defined as the proportion of the SNPs having identical alleles. For the ones with overlap segments shorter than 50%, the similarity is set to 0, since we are particularly interested in extended long homozygosity regions. aTOHs are used to further investigate the allele-specific effects within cTOHs. This process may unveil allele specific effects from the combined effects of matched and non-matched alleles.

### Toh Cluster Tree

In practice, the number of TOHs belonging to different cTOHs may be dramatically different, so is the number of clusters. Therefore, to implement normalized spectral clustering [15], instead of choosing the different number of clusters for each cTOH, we apply the binary tree process. In such a repeated binary spectral clustering algorithm, a hierarchy of clusters is created and represented by a TOH cluster tree. Initially all TOHs in the same cTOH are considered as a root node, and two clusters are created by a binary spectral clustering (i.e., setting the number of clusters ). Then, each of the two clusters, i.e., the leaf node, is further clustered by another binary clustering. This is iterated until a TOH cluster tree is generated where each tree node represents one TOH cluster and it preserves and manages all the clusters, and provides users means of further exploration.

The termination of clustering is based on 1) the depth of the tree, 2) the similarity of each pair of TOHs within each node and 3) the number of TOHs in each node. For example, if the depth of the tree reaches 100, then no clustering will be conducted. Within each node, clustering won’t proceed if the lower quartile of the similarities among the pairs within the node is greater than 0.5 or the number of TOHs within the node is less than 5. Once all clustering is completed, the results can be obtained from the leaves of the binary tree. Among the three criteria, the similarity is relative sensitive, which is based on the summary statistics and cut-off value, in the discussion section, we use the demonstration dataset to illustrate and discuss the best choice of summary statistics and cut-off value. This is a flexible, data-adaptive process, which would meet the various patterns of different cTOHs and will be facilitated by our interactive visualization system.

Starting with the root node for a particular cTOH region, the following normalized spectral clustering algorithm is implemented on the TOHs present in each root/sub-node, assuming there are such TOHs under a particular node:

Step 1: Construct a similarity matrix , where for each pair of TOH and , with the corresponding length of and , and is the length of the two TOHs overlap. Let be the diagonal degree matrix with the diagonal element .

Step 2. Compute the normalized Laplacian , where is an by identity matrix.

Step 3. Let be the matrix with the vectors as columns, which are the first eigenvectors of .

Step 4. Form a normalized matrix from by normalizing the rows to norm 1 by setting . Let be the vector of -th row of , 

Step 5. Cluster the points with the k-means algorithm into clusters . Clusters with .

### Statistical Association Test

A genome-wide case-control analysis was conducted to test the association between surrogate-TOH (i.e., cTOH, gTOH, or aTOH) and the disease. Treating each surrogate-TOH as a genomic variant, which was viewed as a binary variable based on the presence or absence of the surrogate-TOH, a logistic model was fitted by considering disease status as the outcome and each surrogate-TOH as the predictor adjusting for other possible covariates such as age, sex and smoking status. P-values were obtained by Wald tests and odds ratios (95% confidence interval) were calculated through coefficient estimates of the fitted logistic model. For the case of small cell counts (<5), p-values and odds ratios were obtained by Fisher’s exact test.

### “Cgatoh” Software

**Figure 1:**
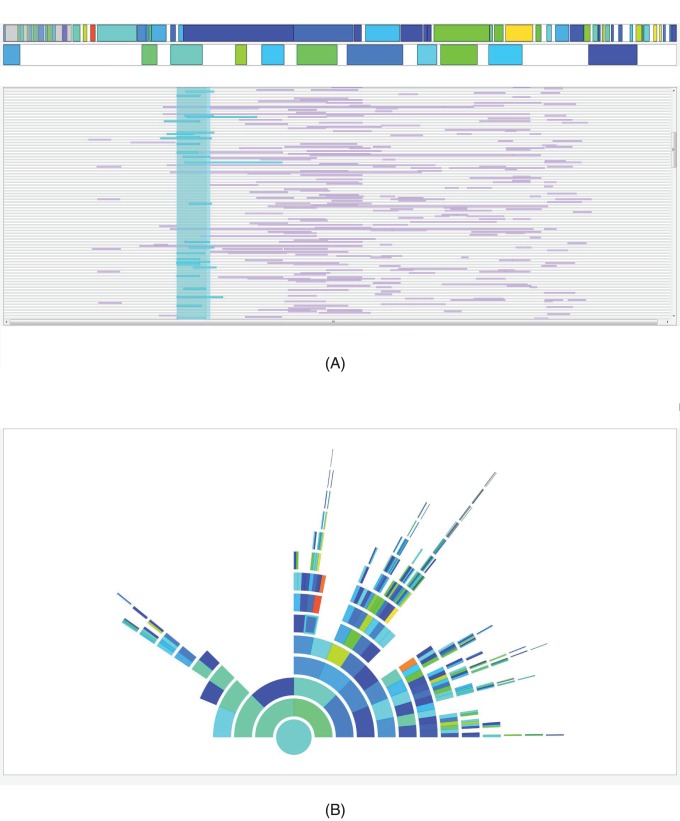
Chromosome and cTOH navigation bars with TOH clusters. (A) TOH cluster explorer. The top panel is the chromosome navigation bar, with a close-up of the cTOH navigation bar below. Clicking a particular cTOH in the cTOH navigation bar will reveal all TOHs within the region in the bottom panel. (B) Navigation ring of TOH cluster tree. The tree ring navigation panel displays the corresponding clustering information based on the binary tree. Ring segments can be clicked on the tree ring navigation panel to highlight the TOHs belonging to that cluster in the TOH view. All colours are based on a heat map that corresponds to p-values of the regions obtained through the association tests.

Based on the proposed algorithm, we developed the cgaTOH software, available at http://www.cs.kent.edu/~;zhao/TOH/. It starts by searching for TOHs and cTOHs, then identifying gTOH and aTOH separately, each accompanied by interactive regional visualization, which results in a software that is more efficient and has increased functionality and flexibility of using appropriate parameters. Identification of TOH, cTOH, gTOH and aTOH is implemented based on C++ with a command line interface with options for choices of parameters, and the visualization interface is created by Qt for the cross-platform User Interface (UI). Figure 1 presents an example of chromosome and cTOH navigation bars with TOH clusters for the sample data set. The input datasets are required to be processed after regular quality control and in the same format as the input files for PLINK [13], i.e.,.ped and.map files. The output files are in tab-delimitated text format, which include both genotype and phenotype information for all TOHs and surrogate-TOHs and are ready for further statistical analysis.

## Results

For illustration purposes, we utilized one of the lung cancer datasets from the GENEVA lung cancer project, PLCO (the Prostate, Lung, Colorectal and Ovarian cancer trial [14], dbGaP Study Accession: phs000207.v1.p1). Study subjects were all from the screening arm and the controls are similar to the cases in age, gender composition and distribution across the recruitment centers. DNA samples were genotyped on the Illumina HumanHap550v3_B arary. Details of the dataset have been previously reported and quality control was done as suggested in the original paper [16]. Samples were screened and selected only if they had a minimum 95% successful genotype call rate. SNPs with minor allele frequencies (MAF) <5%, departures from Hardy-Weinberg equilibrium (at p<0.01) and >5% missingness per SNP, were excluded from further analyses. After quality control, there remained analysable data from 1618 European Americans (788 lung cancer cases and 830 ancestry matched controls), including 967 males and 651 females, 156 non-smokers, 703 previous smokers and 759 current smokers. 514,355 autosomal SNPs were available for subsequent TOH analyses. The mean frequency of homozygous SNP’s was 66.5%.

By using the proposed method and for each subject, on a single chromosome, defining a window of 100 or more consecutive SNPs with homozygosity as a TOH, i.e., , we found 44,725 TOHs in cases and 46,735 TOHs in controls, where average length of TOH is 141.4 SNPs (median = 121 SNPs, 1^st^ quartile = 108 SNPs, 3^rd^ quartile = 145 SNPs) and 886 kb (median = 677.4 kb, 1^st^ quartile = 484.8 kb, 3^rd^ quartile = 956.3 kb). There are 333,861 SNPs with 10 or more TOH calls across the entire sample, 65% of the original pool of SNPs. A total of 890 cTOHs (setting  = 10) were identified across the genome, ranging in length from 100 to 4131 SNPs with median of 215 SNPs (from 141.6 Kb to 34.210 Mb with median of 1064 Kb). Table S1 lists the number of gTOHs and aTOHs detected genome-wide for various thresholds based on different summary statistics (i.e., minimum, lower quartile and mean) of pair-wise similarities within the node. The results in subsequent paragraphs are based on lower quartile of similarity greater than 0.75.

**Figure 2:**
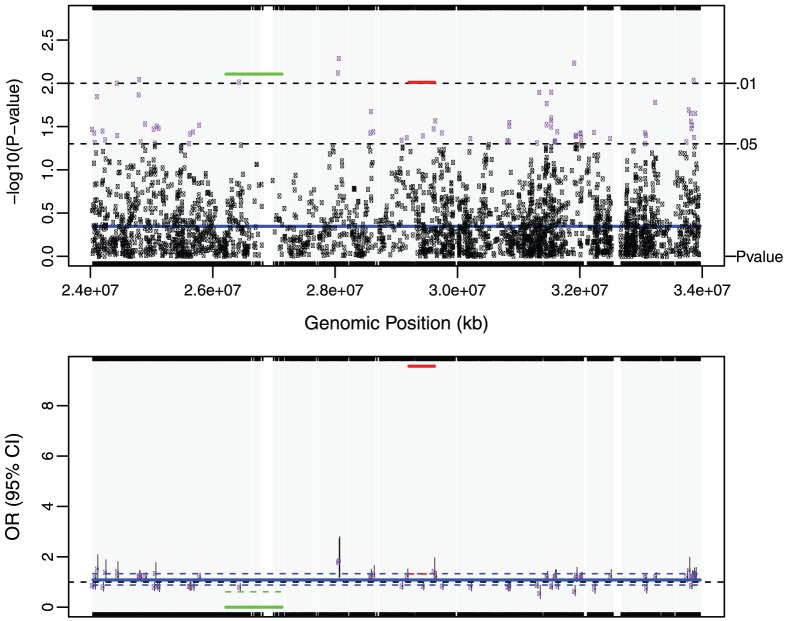
gTOH (rs198845-rs12190473) and aTOH (rs3130778- rs376681) regions associated with lung cancer. (A) –log10 transformed p-values obtained from the association tests. The green line, red line and blue line are the p-values corresponding to gTOH (rs198845-rs12190473), aTOH (rs3130778- rs376681) region and their parent cTOH region, respectively. The purple dots and black dots are p-values<0.05 and > = 0.05 based on single SNP association tests within the same region of the parent cTOH. (B) The corresponding lung-cancer risks as odds ratios (OR) and 95% confidence interval (CI). Green solid line and dash line corresponding to OR and 95%CI for gTOH, while red and blue lines are for aTOH and it’s parent cTOH. The purple dots represent OR for single SNP risk with grey solid vertical lines showing the 95% CIs.

**Figure 3:**
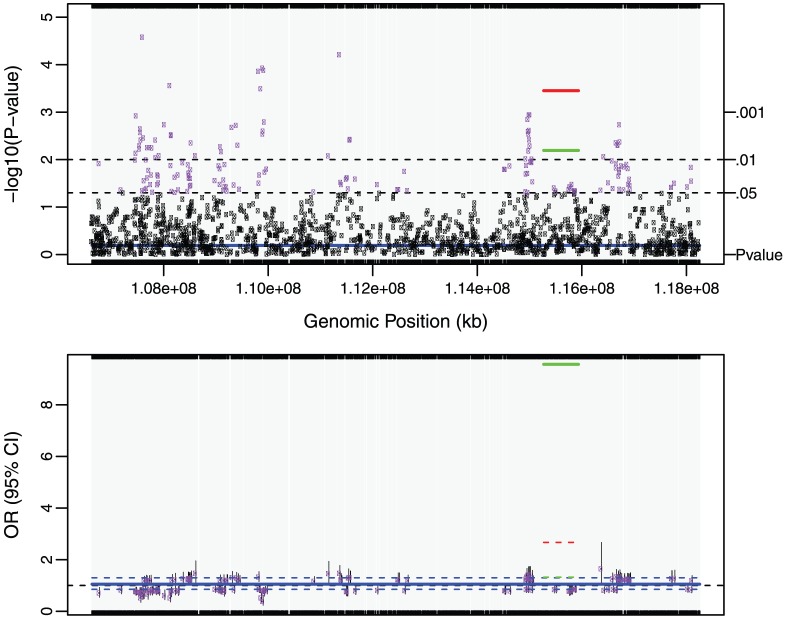
gTOH (rs7812989-rs2884258) and aTOH (rs7812989-rs2884258) regions associated with lung cancer. (A) –log10 transformed p-values obtained from the association tests. The green line, red line and blue line are the p-values corresponding to gTOH (rs7812989-rs2884258), aTOH (rs7812989-rs2884258) region and their parent cTOH region, respectively. The purple dots and black dots are p-values<0.05 and > = 0.05 based on single SNP association tests within the same region of the parent cTOH. (B) The corresponding lung-cancer risks as odds ratios (OR) and 95% confidence interval (CI). Green solid line and dash line corresponding to OR and 95%CI for gTOH, while red and blue lines are for aTOH and it’s parent cTOH. The purple dots represent OR for single SNP risk with grey solid vertical lines showing the 95% CIs.

After controlling for demographics and smoking, we identified 7 cTOHs associated with lung cancer (p-value<0.01) [11]. Three cTOHs were over-represented in cases over controls, whereas 4 were under-represented (see Table 2 in [11]). By using the TOH cluster tree which is accomplished by the repeated binary spectral clustering algorithm, we also detected 7 gTOHs associated with the disease (p-value<0.01), which include 4 case-only and one control-only gTOHs (Table 1), and 5 aTOHs associated with the disease (p-value<0.01), which include 3 case-only (Table 2). We did not observe any TOHs or cTOHs appearing only in either case or control subjects. In addition, there are 6106 gTOHs only present in case subjects, among which 23 gTOHs were found in more than 5 cases (≥0.6% of cases), and 6800 gTOHs only present in control subjects, among which 32 gTOHs were found in more than 5 controls (≥0.6% of cases) (Table S1 in File S2). Twenty-three case-only aTOHs (out of 6442) were found in more than 5 case subjects (≥0.6% of cases) and 23 control-only gTOHs (out of 7279) were found in more than 5 controls (≥0.6% of controls) (Table S2 in File S2). Furthermore, none of the cTOHs, which inhabit the disease-associated gTOHs and aTOHs, was significantly associated with the disease. For example, 6p22.1 and 8q23.3 were identified associated with the lung cancer where both gTOH and aTOH were detected (Figure 2 and [3]), however, the cTOHs where the corresponding gTOHs and aTOHs reside were not detected as significant regions associated with lung cancer, and both regions present prevalent in lung cancer patients. Based on GWAS Catalog [17], 6p22.1 region covers *TRNAA-UGC* which was reported associated with lung adenocarcinoma [16] and 8q23.3 region contains *EIF3H* which was reported associated with colorectal cancer [18],[19].

**Table 1:**
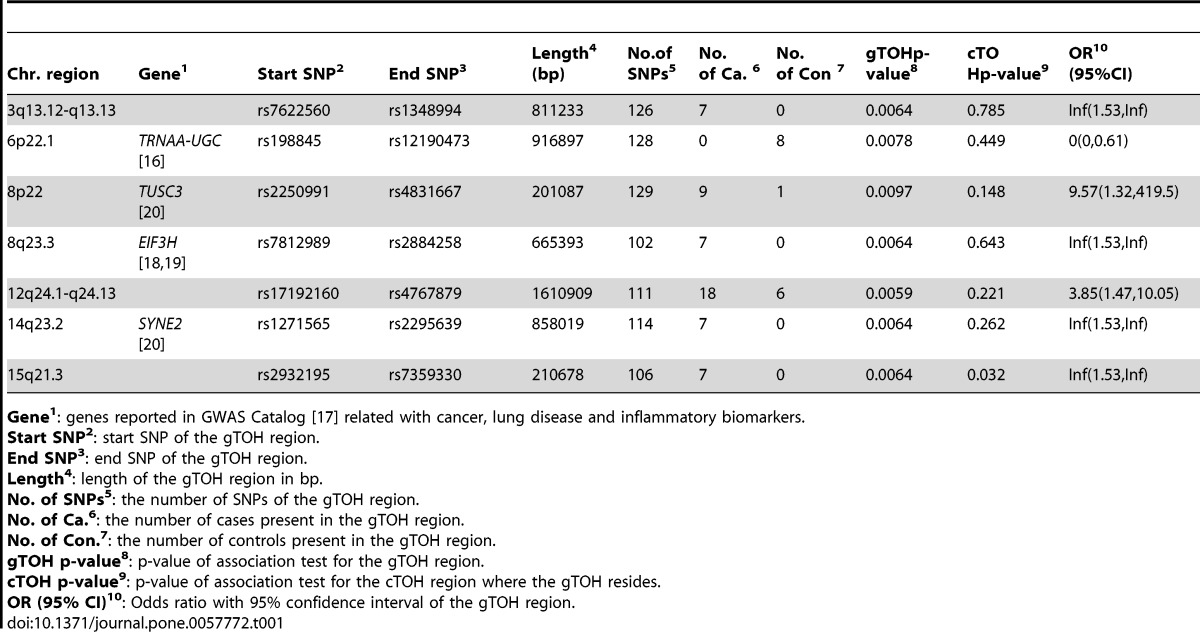
gTOHs significantly associated with lung cancer.

Chr. region	Gene^1^	Start SNP^2^	End SNP^3^	Length^4^(bp)	No.ofSNPs^5^	No.of Ca.^ 6^	No.of Con^ 7^	gTOHp-value^8^	cTOHp-value^9^	OR^10^(95%CI)
3q13.12-q13.13		rs7622560	rs1348994	811233	126	7	0	0.0064	0.785	Inf(1.53,Inf)
6p22.1	*TRNAA-UGC* [16]	rs198845	rs12190473	916897	128	0	8	0.0078	0.449	0(0,0.61)
8p22	*TUSC3* [20]	rs2250991	rs4831667	201087	129	9	1	0.0097	0.148	9.57(1.32,419.5)
8q23.3	*EIF3H* [18],[18]	rs7812989	rs2884258	665393	102	7	0	0.0064	0.643	Inf(1.53,Inf)
12q24.1-q24.13		rs17192160	rs4767879	1610909	111	18	6	0.0059	0.221	3.85(1.47,10.05)
14q23.2	*SYNE2* [20]	rs1271565	rs2295639	858019	114	7	0	0.0064	0.262	Inf(1.53,Inf)
15q21.3		rs2932195	rs7359330	210678	106	7	0	0.0064	0.032	Inf(1.53,Inf)

**Gene^1^**: genes reported in GWAS Catalog [17] related with cancer, lung disease and inflammatory biomarkers.

**Start SNP^2^**: start SNP of the gTOH region.

**End SNP^3^**: end SNP of the gTOH region.

**Length^4^**: length of the gTOH region in bp.

**No. of SNPs^5^**: the number of SNPs of the gTOH region.

**No. of Ca.^6^**: the number of cases present in the gTOH region.

**No. of Con.^7^**: the number of controls present in the gTOH region.

**gTOH p-value^8^**: p-value of association test for the gTOH region.

**cTOH p-value^9^**: p-value of association test for the cTOH region where the gTOH resides.

**OR (95% CI)^10^**: Odds ratio with 95% confidence interval of the gTOH region.

doi:10.1371/journal.pone.0057772.t001

**Table 2:**
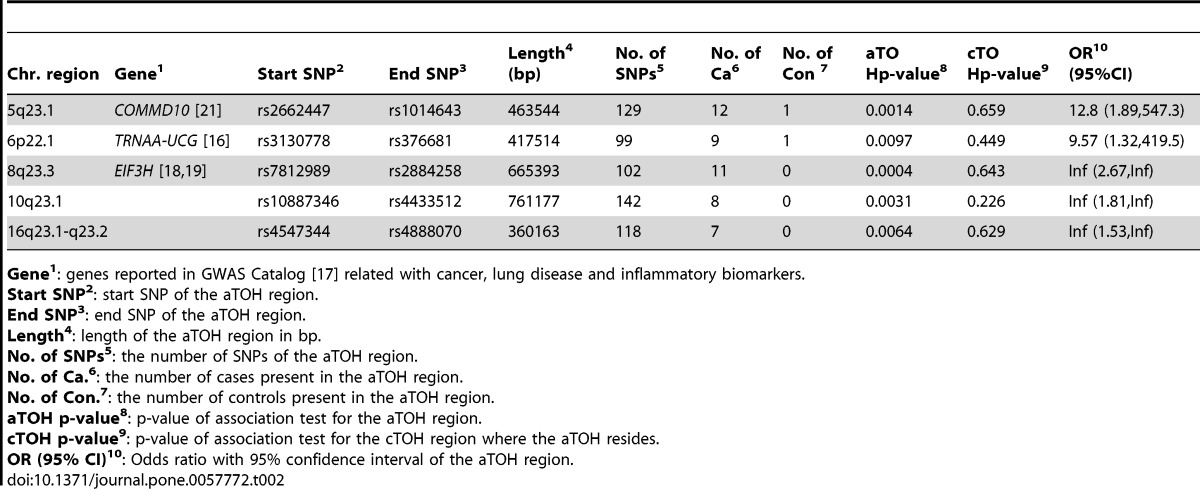
aTOHs significantly associated with lung cancer (PLCO data).

Chr. region	Gene^1^	Start SNP^2^	End SNP^3^	Length^4^(bp)	No. ofSNPs^5^	No. ofCa^6^	No. ofCon^ 7^	aTOHp-value^8^	cTOHp-value^9^	OR^10^(95%CI)
5q23.1	*COMMD10* [21]	rs2662447	rs1014643	463544	129	12	1	0.0014	0.659	12.8 (1.89,547.3)
6p22.1	*TRNAA-UCG* [16]	rs3130778	rs376681	417514	99	9	1	0.0097	0.449	9.57 (1.32,419.5)
8q23.3	*EIF3H* [18],[19]	rs7812989	rs2884258	665393	102	11	0	0.0004	0.643	Inf (2.67,Inf)
10q23.1		rs10887346	rs4433512	761177	142	8	0	0.0031	0.226	Inf (1.81,Inf)
16q23.1-q23.2		rs4547344	rs4888070	360163	118	7	0	0.0064	0.629	Inf (1.53,Inf)

**Gene^1^**: genes reported in GWAS Catalog [17] related with cancer, lung disease and inflammatory biomarkers.

**Start SNP^2^**: start SNP of the aTOH region.

**End SNP^3^**: end SNP of the aTOH region.

**Length^4^**: length of the aTOH region in bp.

**No. of SNPs^5^**: the number of SNPs of the aTOH region.

**No. of Ca.^6^**: the number of cases present in the aTOH region.

**No. of Con.^7^**: the number of controls present in the aTOH region.

**aTOH p-value^8^**: p-value of association test for the aTOH region.

**cTOH p-value^9^**: p-value of association test for the cTOH region where the aTOH resides.

**OR (95% CI)^10^**: Odds ratio with 95% confidence interval of the aTOH region.

doi:10.1371/journal.pone.0057772.t002

## Discussion

The TOH approach differs from single SNP or haplotype association methods employed in most genome-wide association studies, and may be better for identifying chromosomal segments that harbour rare, penetrant recessive loci, which has less ambiguity than generating haplotypes/diplotypes. Another advantage of this approach is that it reduces the number of genomic variants and therefore multiple testing corrections for genome-wide significance. Finding such regions also will be helpful for further investigation (e.g., fine mapping by applying more sophisticated method, such as region-specific analysis), instead of applying sliding windows.

Choosing appropriate parameters is crucial for detection of the long tract of homozygosity regions and further investigation of the disease-associated regions. Our experience suggested that the parameters should be chosen based on knowledge of the data. Lencz and colleagues [1] suggested that the criteria for selecting the number of consecutive SNPs (e.g,  = 100) is based on an order of magnitude larger than the mean haplotype block size found in the human genome. In the PLCO dataset, haplotype block estimation (done by PLINK –blocks) shows the mean haplotype block length as 4.4 SNPs, which suggests that the minimum length of TOH should be set at least 45 SNPs. Furthermore, the PLCO dataset has a 0.665 mean probability of being homozygous across all 514,355 SNPs belonging to 22 autosomal chromosomes for 1618 subjects, and following Lencz et al. [1], by ignoring regional variability in LD and heterozygosity, the likelihood of observing L consecutive chance of homozygosity can be described in the formula of . When  = 58, this likelihood is less than 5%, which means that a minimum length of  = 58 independent SNPs would have less than 5% likelihood of generating TOHs. However, this assumes complete independence among the SNPs, so Lencz et al [1] considered using a correction by using the tag groups to account for LD among the SNPs. In the PLCO dataset, 323,374 separable tag groups were found by genome-wide identification of tag SNPs within windows of 250 kb and >0.8 (by PLINK –show-tags), which could represent 63% of the total number of SNPs. Thus, adjusting the run size to 92 ( = 58/0.63) SNPs could approximate the same degrees of freedom as 58 independent SNP calls.

Here, we also recommend an empirical way to consider the appropriate range of . Depending on the region or chromosome, the fixed number of SNPs may either cover a larger or shorter genomic region, which could be due to several reasons, e.g., recombination or LD hotspots that governed the SNP selection process. The pervasiveness of LD ensures that we can expect many small TOHs in human populations, while we are looking for meaningful long tracts of homozygosity. A survey of the length of stretches of loci in strong LD (also referred to as a haplotype block) across the human population shows that very few will reach over 20 SNPs (a hundred kb) in length. For the PLCO dataset, we observed the average length of a haplotype block is 4.4 SNPs, with 99% quantile of the haplotype block length being 16 SNPs. To eliminate those shorter TOHs generated from local LD, we are studying on the TOHs with lengths greater than 16 SNPs, which can be obtained by implementing the first step of the cgaTOH software and setting  = 17 SNPs. Considering the distribution of the length of all those TOHs with length greater than 17 SNPs, 95% and 99.9% quantiles of those TOHs length are 72 and 112 SNPs, respectively. Therefore, to allow meaningful long tracts of homozygosity (longer than haplotype block) with small likelihood (<5%) but avoid only capturing very rare occurrences of TOHs (<0.1%), the length of TOHs should be somewhere between 72 and 112 SNPs.

Lencz et al [1] stated the selection of reflects biological meaning and statistical feasibility, where was set  = 10. Across from 50 to 120 SNPs under different choices of , we further tabulate different characteristics of detected cTOHs, i.e., the median number of subjects belonging to a single cTOH, the median length of cTOHs and the median number of observed cTOHs. When  = 90 to 120, the median number of subjects belonging to each cTOH is relative stable within the range of 50 to 100 (3% to 6% of the total number of samples) (Figure S1 A in File S1), suggesting a narrower range for the choice of , i.e., , which is consistent with the information provided by the median cTOH length plot (Figure S1 B in File S1). The plot of the median number of observed cTOHs indicates that with a relatively large (e.g.,  = 30, 40 and 50), the number of observed cTOHs changes dramatically, in contrast, the plateau of the curves of smaller covers larger range of (Figure S1 C in File S1), which implies smaller has less effect on the number of observed cTOHs across a certain range of . Considering  = 90∼110, the curves of  = 10 provides relative a stable profile. Therefore, for the illustrative PLCO dataset,  = 100 and  = 10 would be an appropriate choice.

In summary, a relatively large (>120) is not suggested, unless extremely long tracts are expected, while small is also not recommended to avoid capturing haplotype blocks instead of a *bona fide* TOH. One is recommended to start with analysis of haplotype blocks and then to check the distribution of the TOH length. Choosing within a reasonable range will help one to find more significant regions without losing the extremely long tracts. Choice of is less critical here, so one could think of as a reasonable percentage of the whole sample size to retain the power for further (statistical) analysis among biologically meaningful TOHs.

The highlight of the current method is identifying proximally-bounded gTOHs and allelically-matched aTOH regions, which do not exist in the existing software. Individuals harbouring gTOH are most likely going to cladistically group together and will share similar recent ancestral alleles. The main advantage of aTOH is that the distribution and association of similar allelic pairs may shed light in the similarity of genetic background in the region across cases and controls compared within the cTOH. gTOH and aTOH hence provide plausible biologically meaningful analysis. In the illustrative PLCO datasets, some of the gTOHs and aTOHs are significant while their respective parent cTOH are not. Introducing the concept of surrogate-TOH is able to provide genetic background or ancestry related information hence a biological meaningful association with phenotype.

In the iterative binary spectral clustering, the most important stopping rule is the similarity of each pair of TOHs within each node greater than a threshold. Table S1 and S2 in File S2 summarize the number of gTOHs and aTOHs detected under different criteria of similarity based on various summary statistics, respectively. Generally speaking, the higher the criterion is, the more clusters (gTOH and aTOH) will be detected, resulting smaller cluster size. Using average similarity is relative too loose, whereas minimum similarity is relative too stringent. Ideally, we are expecting all similarity is 100%, i.e., the minimum pair-wised similarity should be 100%, which is very strict criterion, and not feasible for our ultimate goal of association tests. Therefore, we suggest lower quartile of all pair-wised similarities greater than 75% within a node, thus allows a certain number of heterozygotes and/or missing genotypes which may break the long tracts of homozygosity and also reduces bias due to outliers or sampling errors but still be consistent with the majority (75%) within each node. The higher similarity criterion (>0.95) is not favourable, which tends to have more clusters with less subjects in each cluster, thus reduces the power of statistical testing. Unless very strict long extended homozygosity regions are expected, minimum similarity >0.95 won’t be applied.

In summary, we propose a new algorithm to identify various patterned clusters of SNPs demonstrating extended homozygosity from individual TOH to surrogate-TOH, thus one can observe different aspects of the multi-faceted characteristics of TOHs and detect more stringent long extended homozygosity regions. The developed software integrates all the above components with the genome-wide visualization system, allowing intuitive navigation and exploration of the data to aid in further downstream analysis.

## Supporting Information

File S1 The influence of and on the results of cTOH. (A) The median number of subjects belong to each cTOH versus the minimum TOH length (L) under different choices of the minimum number of individuals (n) containing such TOH calls. (B) The median cTOH length versus the minimum TOH length (L) under different choices of the minimum number of individuals (n) containing such TOH calls. (C) The total number of cTOHs observed versus the minimum TOH length (L) under different choices of the minimum number of individuals (n) containing such TOH calls.
(EPS)

File S2 Summary of the number of gTOHs detected under different criteria of similarity. Table S2: Summary of number of aTOHs detected under different criteria of similarity.
(DOC)
