# Supplementary material for: cgaTOH: Extended Approach for Identifying Tracts of Homozygosity
Source: PLoS One. 2013 Mar 1;8(3):e57772. doi: 10.1371/journal.pone.0057772 (PMC3585782; doi:10.1371/journal.pone.0057772)
Supplement: File S2 — Summary of the number of gTOHs detected under different criteria of similarity. Table S2: Summary of number of aTOHs detected under different criteria of similarity. (DOC) [file pone.0057772.s002.doc]

Supplementary Table 1: Summary of the number of gTOHs detected under different criteria of similarity

| Summary statistics | Similarity criterion | No. of  detected gTOHs | No. of  case-only gTOHs | No. of  control-only gTOHs | No. of significant gTOHs |
| --- | --- | --- | --- | --- | --- |
| Minimum | 0.50 | 20410 | 5313 (35) | 6010 (39) | 8 |
|  | 0.75 | 24762 | 6871 (32) | 7518 (38) | 7 |
|  | 0.95 | 31118 | 9063 (12) | 9839 (22) | 2 |
| Lower quartile | 0.50 | 13642 | 3177 (22) | 3701 (35) | 9 |
|  | 0.75 | 22639 | 6106 (23) | 6800 (32) | 7 |
|  | 0.95 | 30801 | 8969 (17) | 9730 (17) | 1 |
| Mean | 0.50 | 3728 | 479 (13) | 652 (12) | 8 |
|  | 0.75 | 13789 | 3206 (17) | 3821 (25) | 8 |
|  | 0.95 | 29343 | 8346 (27) | 9166 (28) | 4 |

Summary statistics: the summary statistics that summarizes all pair-wise similarities within each cluster.

Similarity criterion: the criterion used in normalized spectral clustering.

No. of detected gTOHs: the number of gTOHs detected in whole-genome

No. of case-only gTOHs: the number of gTOH detected only in lung cancer patients, with the number of gTOH detected in more than 4 patients in parentheses, but not in controls.

No. of control-only gTOHs: the number of gTOH detected only in controls, with the number of gTOH detected in more than 4 controls in parentheses, but not in patients.

No. of significant gTOHs: the number of gTOHs significantly associated with the disease based on p-value<0.01.

Supplementary Table 2: Summary of number of aTOHs detected under different criteria of similarity

| Overlap percentage | Summary statistics | Similarity criterion | No. of  detected aTOHs | No. of  case-only aTOHs | No. of control-only aTOHs | No. of significant aTOHs |
| --- | --- | --- | --- | --- | --- | --- |
| 0.50 | Minimum | 0.50 | 22001 | 5873 (31) | 6649 (27) | 5 |
|  |  | 0.75 | 25505 | 7334 (21) | 8170 (21) | 8 |
|  |  | 0.95 | 28748 | 8680 (27) | 9526 (24) | 3 |
|  | Lower quartile | 0.50 | 16626 | 3921 (23) | 4582 (23) | 5 |
|  |  | 0.75 | 23256 | 6442 (23) | 7279 (23) | 5 |
|  |  | 0.95 | 27873 | 8242 (19) | 9177 (9) | 1 |
|  | Mean | 0.50 | 6055 | 1089 (20) | 1357 (15) | 10 |
|  |  | 0.75 | 15221 | 3653 (20) | 4334 (20) | 3 |
|  |  | 0.95 | 25642 | 7357 (15) | 8252 (22) | 3 |
| 0.75 | Minimum | 0.50 | 24436 | 6796 (16) | 7828 (26) | 3 |
|  |  | 0.75 | 24436 | 6796 (16) | 7828 (26) | 3 |
|  |  | 0.95 | 28431 | 5313 (19) | 6010 (27) | 3 |
|  | Lower quartile | 0.50 | 19492 | 5118 (21) | 5936 (26) | 5 |
|  |  | 0.75 | 22561 | 6162 (26) | 6992 (28) | 5 |
|  |  | 0.95 | 27587 | 8195 (22) | 9240 (23) | 1 |
|  | Mean | 0.50 | 10684 | 2586 (27) | 2984 (25) | 7 |
|  |  | 0.75 | 16386 | 4321 (19) | 4990 (27) | 5 |
|  |  | 0.95 | 25011 | 7193 (15) | 8121 (24) | 1 |
| 0.95 | Minimum | 0.50 | 27785 | 5313 (30) | 6010 (34) | 6 |
|  |  | 0.75 | 27785 | 5313 (30) | 6010 (34) | 6 |
|  |  | 0.95 | 27785 | 5313 (30) | 6010 (34) | 6 |
|  | Lower quartile | 0.50 | 23921 | 7243 (25) | 7595 (49) | 3 |
|  |  | 0.75 | 25746 | 7821 (26) | 8170 (44) | 2 |
|  |  | 0.95 | 27597 | 8530 (27) | 8967 (41) | 5 |
|  | Mean | 0.50 | 16208 | 4952 (18) | 4981 (40) | 4 |
|  |  | 0.75 | 20987 | 6424 (17) | 6658 (34) | 4 |
|  |  | 0.95 | 26428 | 5313 (22) | 6010 (40) | 5 |

Overlap percentage: the minimum percentage that the overlap of the pairs covers each individual TOH.

Summary statistics: the summary statistics that summarizes all pair-wise similarities within each cluster.

Similarity criterion: the criterion used in normalized spectral clustering.

No. of detected aTOHs: the number of aTOHs detected in whole-genome

No. of case-only aTOHs: the number of aTOH detected only in lung cancer patients, with the number of aTOH detected in more than 4 patients in parentheses, but not in controls.

No. of control-only aTOHs: the number of aTOH detected only in controls, with the number of aTOH detected in more than 4 controls in parentheses, but not in patients.

No. of significant aTOHs: the number of aTOHs significantly associated with the disease based on p-value<0.01.
